# Topographic correspondence between retinotopic and whisker somatosensory map in mouse higher visual area and its development

**DOI:** 10.3389/fncir.2025.1552130

**Published:** 2025-09-02

**Authors:** Hanaka Matsumoto, Tomonari Murakami, Kenichi Ohki

**Affiliations:** ^1^Department of Physiology, Graduate School of Medicine, The University of Tokyo, Tokyo, Japan; ^2^Institute for AI and Beyond, The University of Tokyo, Tokyo, Japan; ^3^International Research Center for Neurointelligence (WPI-IRCN), The University of Tokyo, Tokyo, Japan

**Keywords:** topographic correspondence, retinotopy, somatotopy, multisensory integration, spontaneous cortical activity, development, rostrolateral area, posterior parietal cortex

## Abstract

Aligning the topography maps of different sensory modalities in the brain is considered to be important for the unified perception of multiple sensory modalities. In mice, the superior colliculus receives both visual and whisker-related somatosensory information with the topographical correspondence between retinotopy and somatotopy. However, it remains unclear whether topographical correspondence between retinotopy and whisker somatotopy exists in the higher association cortex, and if so, how this functional organization is formed during development. Here, we conducted wide-field calcium imaging and revealed retinotopic and somatotopic correspondence in the rostrolateral area (RL), one of the higher visual areas. The retinotopic map demonstrates that RL is divided into two distinct subregions, anterior and posterior parts of RL (RLa and RLp). We further found a rough topographic correspondence between retinotopy and whisker somatotopy only in RLa, but not in RLp, Lastly, to test whether this topographic correspondence exists before eye-opening, we performed functional connectivity analysis of spontaneous cortical activity recorded from developing mice. We discovered that the topographical correspondence between retinotopy-like and somatotopy-like structures in RLa already existed before eye-opening, on postnatal day 10–11. Because spatially corresponding multisensory inputs are likely quite weak before eye-opening, these results in developing mice suggest that the initial formation of topographic correspondence between retinotopy and whisker somatotopy in the higher association cortex does not depend on spatially corresponding multisensory input experiences.

## 1 Introduction

Sensory information received by peripheral organs passes through the thalamic nuclei and midbrain to reach cortical regions specialized for each sense. For example, visual information is processed in the visual cortex of the occipital lobe, auditory information in the auditory cortex of the temporal lobe, and tactile information in the somatosensory cortex of the parietal lobe. Through this sensory processing pathway, topographical structures are maintained in each modality, such as the visual field (retinotopy; Seabrook et al., 2017), body parts (somatotopy; Staiger and Petersen, 2021) or sound frequency (tonotopy; Schreiner and Winer, 2007). The sensory features extracted from different modalities go under integration, referred to as multisensory integration, to help detect or accelerate the processing of ambiguous inputs (Stein and Stanford, 2008). Multiple sensory information in the natural world occurs at spatially close locations due to the same cause, such as an insect’s shape and the sound it makes (Meredith and Stein, 1986). Thus, aligning spatial location codes of the external world, or topographic structures across sensory modalities should be necessary for multisensory integration. Furthermore, the alignment of topographic structures across modalities requires some developmental mechanisms.

Multisensory integration is known to take place in a variety of brain areas, including the superior colliculus (SC) (Meredith and Stein, 1986), striatum (Reig and Silberberg, 2014), and associative cortex (Olcese et al., 2013). Multisensory integration and the alignment of topographic maps have been well studied in SC, and optic tectum (OT), which is a homologous structure to mammal SC (Cang and Feldheim, 2013). For example, in mice, it has been known for a long time that visual and whisker receptive fields correlate in SC (Drager and Hubel, 1975a, 1975b). In the OT of barn owls, there are neurons that respond to both visual and auditory stimulations with the spatial alignment between the visual and auditory receptive fields (Knudsen, 1982). Such a topographic correspondence at SC or OT has been reported not only in mice and barn owls but also in ferrets (King, 1993, 2004), cats (Meredith and Stein, 1996), and rhesus monkeys (Wallace et al., 1996).

In the cortical associative regions, topographical correspondence or spatial congruence of receptive fields between different modalities was reported, as in SC and OT. For example, rough spatial congruence between the visual and somatosensory information has also been reported in the human parietal area (VIP+), which is thought to be homologous to the ventral intraparietal area (VIP) of the macaque monkeys (Huang et al., 2017). In the VIP of the macaque monkey, multisensory integration occurs the most when the receptive fields for visual and somatosensory stimulation are spatially congruent (Avillac et al., 2007). In rats, the border area between the occipital lobe and the parietal lobe is reported to have cells with partly overlapping receptive fields between visual and somatosensory modalities, though the rear parts of the body (the hindlimb, hip, and tail) were used as the targets of somatosensory stimulation and these locations were hardly in the visual field of the animals (Wallace et al., 2004). In mice, an area called the rostrolateral area (RL), which is sometimes referred to as a part of the posterior parietal cortex (PPC) (Glickfeld and Olsen, 2017; Lyamzin and Benucci, 2019), not only visual information but also whisker somatosensory information is processed to integrate them (Olcese et al., 2013). However, it is unclear how well retinotopic and somatotopic organizations correspond in RL (but see Olcese et al., 2013).

If retinotopic and somatotopic maps of RL are aligned in adult mice, what would be the developmental mechanisms for forming this topographical correspondence? One possibility is that the correspondence is formed based on extrinsic sensory inputs from multiple modalities that are spatially congruent. It is known that the formation of an auditory map in barn owl OT is inhibited when raised in an environment without acoustic cues about location (Efrati and Gutfreund, 2011). Moreover, in OT of barn owls (Knudsen et al., 1991) and SC of ferrets (King and Carlile, 1993), it is reported that when the animals are reared with disrupting the visual inputs, the auditory map gets misaligned with the visual map, suggesting that visual and auditory experiences are important for aligning receptive fields of both modalities. In this way, repeated exposure to temporally and spatially congruent multimodal stimuli may be necessary for aligning retinotopy and somatotopy in the cortex.

Another possibility is that topographic organization is formed depending on the intrinsic programs, such as spontaneous neural activity or axon guidance molecules. Previous studies have reported that topographic structures are formed by intrinsic programs, particularly spontaneous activity from peripheral sensory receptors, in various sensory areas including the visual (Torborg and Feller, 2005; Cang et al., 2005a; Xu et al., 2011), somatosensory (Allène et al., 2008; Erzurumlu and Gaspar, 2012), and auditory cortices (Clause et al., 2014). Regarding the visual system development, a recent study has shown that retinotopic information embedded in retinal spontaneous activity propagates to both primary (V1) and higher-order visual areas (HVAs) and that spontaneous activity from the retina is essential to form the retinotopic cortical connections between V1 and HVAs (Murakami et al., 2022). Because the topographic corticocortical connections at least in the visual pathway require spontaneous activity, it is likely that the intrinsic spontaneous activity from multimodal sensory pathways is employed in topographical correspondence development between different modalities, although the molecular control by axon guidance molecules may also contribute to the development of topographic alignment.

The present study aims to investigate to what extent visual and whisker topographic structures correspond in the HVA of adult mice and to test whether the topographic correspondence is already observed during development before eye-opening. Questions related to the latter purpose are: does the initial formation process of rough multimodal topographic correspondence in RL depend on spatially congruent extrinsic sensory inputs from different modalities? Or does it depend on intrinsic programs, such as spontaneous activity as is shown for the development of the unimodal system (Murakami et al., 2022)? If the former possibility is correct, the approximate correspondence between retinotopic and somatotopic maps will not be formed until eye-opening. On the other hand, if the latter is true, a prototype of retinotopy and somatotopy, and even their correspondence, can be observed in the intrinsic spontaneous activity patterns. To address these questions, we analyzed visual and whisker topographic structures in various cortical areas including RL using wide-field calcium imaging.

## 2 Results

### 2.1 RL is divided into two subregions based on the retinotopic map

**Figure 1:**
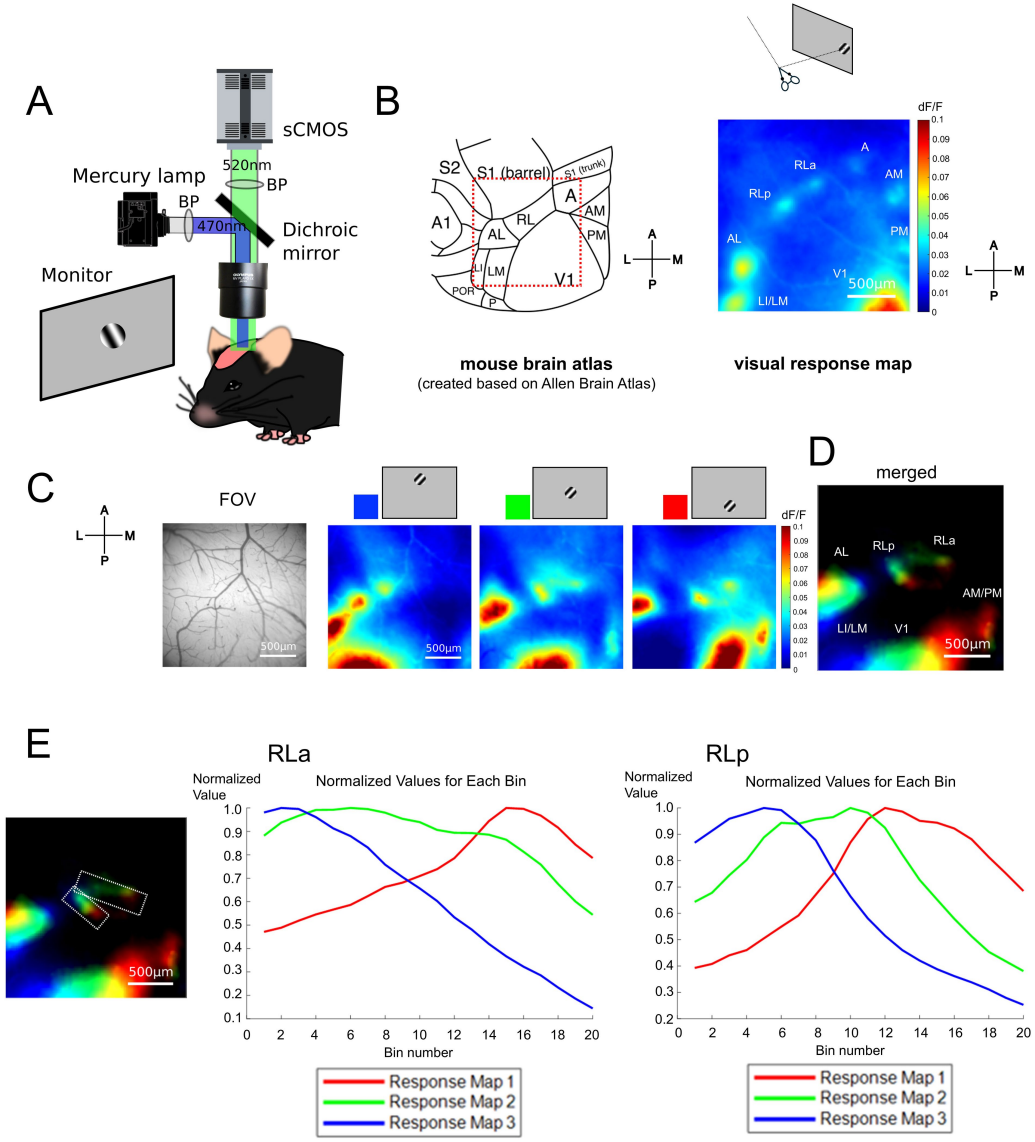
RL was divided into two subregions based on the retinotopic map. **(A)** Experimental setups for wide-field calcium imaging in adult mice. **(B)** (Left) A schema of the mouse cortical atlas based on the Allen Brain Atlas (https://connectivity.brain-map.org/3d-viewer) (Lein et al., 2007). The squared region surrounded with red dotted lines shows the field of view (FOV) roughly corresponding to the visual response map (right). (Right) A visual response map obtained by presenting the drifting grating stimulus at the temporal visual field. Scale bar: 500 μm. A1, primary auditory area; A, anterior area; AL, anterolateral area; AM, anteromedial area; LI, laterointermediate area; LM, lateromedial area; P, posterior area; PM, posteromedial area; POR, postrhinal area; RL, rostrolateral area; S1 (barrel), barrel cortex of the primary somatosensory area; S1 (trunk), trunk region of the primary somatosensory area; S2, secondary somatosensory area; V1, primary visual area. **(C)** The FOV and visual response maps, showing the retinotopic organization. The visual stimuli were presented from the upper to the lower visual field. The colors at the top of the maps (blue, green, and red) correspond to the color coding in **(D)**. Scale bar: 500 μm. **(D)** Retinotopic organization of visual areas. Scale bar: 500 μm. **(E)** Difference of peak responsive region in RLa and RLp. (Left) Retinotopy map which is identical to **(D)**, on which the region of interest (ROI) of RLa and RLp are overlaid (white dashed lines). Scale bar: 500 μm. (Middle and right) Plots of normalized dF/F of RLa and RLp, respectively. The ROIs for RLa and RLp were segmented into 20 bins along the upper-lower axis (i.e., the longer side of the parallelograms). Within each bin, the normalized dF/F was calculated for each map corresponding to blue, green, and red channels respectively, by dividing the original dF/F with the absolute peak value. The x-axis indicates the bin number. Bin number from 1 to 20 corresponds to the upper to lower in the axis. The y-axis indicates the normalized dF/F. Blue, green, and red correspond to the response map with upper to lower visual stimuli.

To obtain the retinotopic map, we performed wide-field calcium imaging on Thy1-GCaMP6s mice which express GCaMP6s in the excitatory neurons (see Methods, Figure 1A). We observed some responsive spots around V1 in the response map to the stimulus presented at approximately 20 degrees in elevation and 100 degrees in azimuth under a coordinate system centered on the animal (Figure 1B). By comparing the response map with a representative anatomical study (Wang and Burkhalter, 2007) and the widely accepted mouse brain atlas (Allen Mouse Brain Atlas; Lein et al., 2007),^1^ we found that each responsive spot seemed to correspond to each HVA in the atlas except for area RL. As observed in previous studies, only a single responsive spot was observed in anatomically determined HVAs such as areas LI (laterointermediate area)/LM (lateromedial area), AL (anterolateral area), A (anterior area), AM (anteromedial area), and PM (posteromedial area), while there were two clearly separate responsive spots within the RL (Figure 1B). We designated the anterior part of RL as RLa and the posterior part as RLp.

To examine whether RLa and RLp each have a distinct retinotopic structure, we obtained three response maps to stimuli presented at three different locations from the upper to the lower visual field (Figure 1C) and merged them to create a retinotopic map (Figure 1D) and found independent retinotopic structures at RLa and RLp. We used five mice, and all of them showed the segregation between RLa and RLp and individual retinotopic structures for these two areas in the retinotopic map (Supplementary Figure 1). Next, we examined whether the response profiles to stimuli presented at three different locations from upper to lower visual field differed in RLa and RLp, which were determined based on retinotopy (Figure 1E, left panel). Each region was segmented into 20 bins and the averaged dF/F within each bin was calculated and the normalized values of dF/F divided by the absolute peak value of dF/F was plotted (Figure 1E). These plots show that the three peaks of visual response in RLa and RLp are sequential, indicating that RLa and RLp have each retinotopic structure and are distinct cortical areas.

We demonstrated that RL, which has been thought of as one region (Wang and Burkhalter, 2007; Garrett et al., 2014), was actually divided into two different areas based on the retinotopic structure (Figures 1C–E). Since RL is known to process both visual and somatosensory whisker information (Olcese et al., 2013), we next investigated the functional difference between RLa and RLp in the response to visual, whisker, and combined stimuli. To address this, we compared the responses to these three conditions in RLa and RLp [AL/LM (lateral HVAs) and V1 for comparison] (Supplementary Figure 2). RLa showed a response not only to visual stimuli but also to whisker stimuli (Supplementary Figures 2A,E) while the other visual areas showed only a little response to whisker stimuli (Supplementary Figures 2B–D, 2F–H). These results suggest that RLa and RLp are functionally different regarding the response to whisker stimulation.

### 2.2 Somatotopy of whiskers in RLa and its alignment with retinotopy

**Figure 2:**
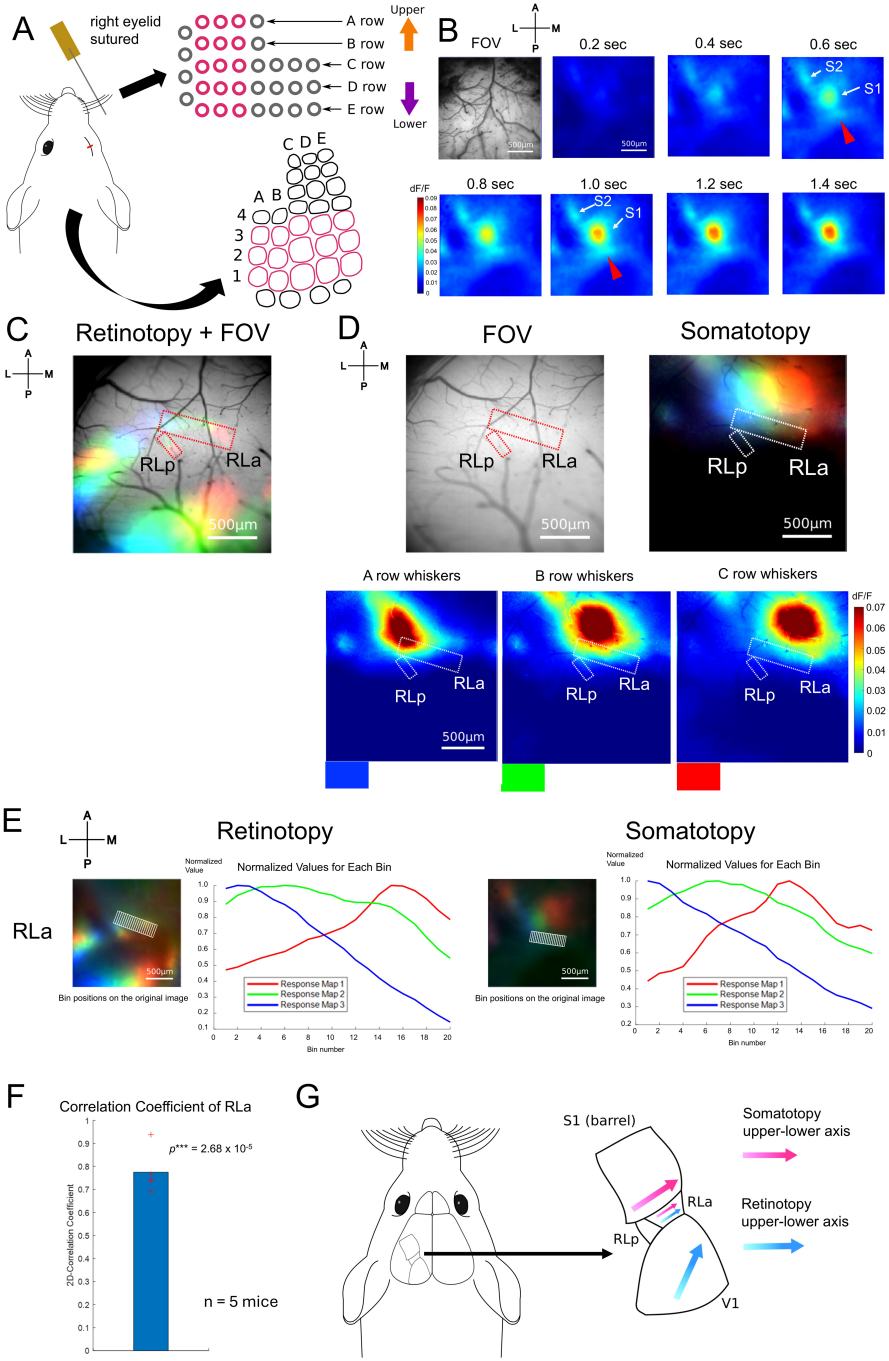
Somatotopy of whiskers in RLa and topographic overlap. **(A)** Whisker stimulating setups. The pink lines indicate the positions of retained whiskers and the the corresponding S1 barrel areas (see Methods). The right eyelid was sutured to avoid the effect of light as much as possible. A piezoelectric device with around 10 cm wire attached was used to stimulate two to three whiskers in the same row at the same time. **(B)** Example of cortical response upon the stimulation of B-row whiskers after a stimulus onset (0 s). Scale bar: 500 μm. **(C)** Retinotopy overlaid on the field of view (FOV). The positions of RLa and RLp were decided based on the retinotopic map (red dotted lines). Scale bar: 500 μm. **(D)** (Top, left) The positions of RLa and RLp overlaid on FOV. The RLa and RLp position were transformed from those from the retinotopic map to match the somatotopy FOV based on the vessel patterns (see Methods). (Bottom) The whisker response maps showing the somatotopic organization. Whisker stimulations were presented to A-row, B-row, and C-row whiskers. The colors at the bottom of the maps (blue, green, and red) correspond to the color coding in the merged image (Top, right). Dashed white lines indicate the ROIs for RLa and RLp, which were identified based on the retinotopic maps. Scale bar: 500 μm. **(E)** The shift of visual and whisker-responsive regions depending on the stimulus position in RLa. Scale bar: 500 μm. dF/F was normalized by dividing the original dF/F with the absolute peak value in each color. The x-axis indicates the bin number and the y-axis indicates the normalized dF/F. The retinotopy and somatotopy were obtained from the same animal but from slightly different FOVs. Eyes were open when retinotopic mapping was performed, and the right eyelid was sutured when somatotopic mapping was performed. The ROI for RLa in the retinotopic map was converted to match the FOV position of the somatotopic map based on vessel patterns (see Methods). **(F)** Quantification of topographic correspondence between retinotopy and somatotopy in RLa by 2D-correlation (*n* = 5 mice). The red cross marks represent the 2D-CC from each mouse. RLa showed 2D-CC that was significantly higher than zero (0.775 ± 0.0951) (mean ± standard deviation (SD); one-sample one-sided *t*-test; *p* = 2.68 × 10^−5^ < 0.025). ***p-value smaller than the significance level at 0.001. **(G)** Summary figure for adult mice. RL was divided into two subregions, RLa and RLp, based on the retinotopic organization (Figure 1). RLa showed rough topographical correspondence along the upper-lower axis for both retinotopy and somatotopy **(E,F)**.

To obtain the somatotopic map, we recorded the response to whisker stimulation using wide-field calcium imaging. For whisker stimulation, two or three whiskers within each row of A1–A3, B1–B3, C1–C3, D1–D3, and E1–E3 contralateral to the imaged hemisphere were stimulated by metal wire attached to a piezoelectric bimorph bender (see Methods, Figure 2A). During whisker stimulation experiments, the eyelid contralateral to the imaged hemisphere was sutured to minimize the effect of any visual stimulation. We found that the primary somatosensory area (S1), the secondary somatosensory area (S2), and a region posterior to S1 responded to B-row whisker stimulation (Figure 2B). We then examined whether this region corresponded to RLa and had a somatotopic structure according to the whisker position. Overlaying the RLa and RLp positions identified based on the retinotopic map (dashed red lines, Figure 2C) onto the whisker response maps (dashed white lines, Figure 2D right), we found that RLa responded to whisker stimulation, while RLp almost did not in all five mice used for the experiments (Figure 2D, Supplementary Figure 3). We also observed a shift in responding regions from the lateral to the medial when the stimulus position was changed from A-row to C-row in five mice, including the one in the main figure (Figure 2D, Supplementary Figure 3). Of note, although RLa was mostly activated by A-, B- and C-row whisker stimulation, response to A- or C- row whiskers was sometimes weak at the individual level (Figure 2D, Supplementary Figure 3).

To quantify the topographical alignment, we obtained profiles of visual and whisker responses using the same procedure (see Methods) as in Figure 1E and calculated their correlation coefficient. Then, we calculated the two-dimensional correlation coefficient (2D-CC) between normalized dF/F values of the retinotopic and somatotopic maps in RLa (Figure 2E). 2D-CC of RLa was revealed to be significantly higher than zero (0.775 ± 0.0951) [mean ± standard deviation (SD); one-sample one-sided *t*-test; *p* = 2.68 × 10^−5^ < 0.025] (Figure 2F), indicating that retinotopic and somatotopic structures of RLa were roughly aligned along the upper-lower axis in the stimulus space (Figure 2G).

Next, we performed two-photon calcium imaging using the same whisker-stimulation protocols to test that somatotopy in RLa is due to the activity of neurons in RLa, not solely due to axonal projections from S1 (Supplementary Figure 4). We sutured the eyelid contralateral to the imaged hemisphere as we did in Figures 2A–D to minimize the effect of any visual stimulation. We found that some neurons in RLa responded to whisker stimulation (Supplementary Figures 4B,C, top), and responding positions in RLa shifted from lateral to medial as the stimulated whiskers changed from A row to C row (from upper to lower) (Supplementary Figure 4C, top). To analyze individual neural activity, we detected neurons using template matching on a local correlation image (see Methods). Responsive neurons were identified as those exceeding the threshold (≥ 3% dF/F and *p* < 0.01, *t*-test). Binary maps of responsive neurons revealed a gradual shift in the spatial distribution of activated neurons in RLa corresponding to the position of the stimulated whisker (Supplementary Figure 4C, bottom; Supplementary Figure 4D). These results suggest that somatotopy observed in RLa using wide-field imaging is, at least in part, due to neural activity within RLa.

### 2.3 Retinotopy-like and somatotopy-like patterns of ongoing activity in the developing mouse cortex

**Figure 3:**
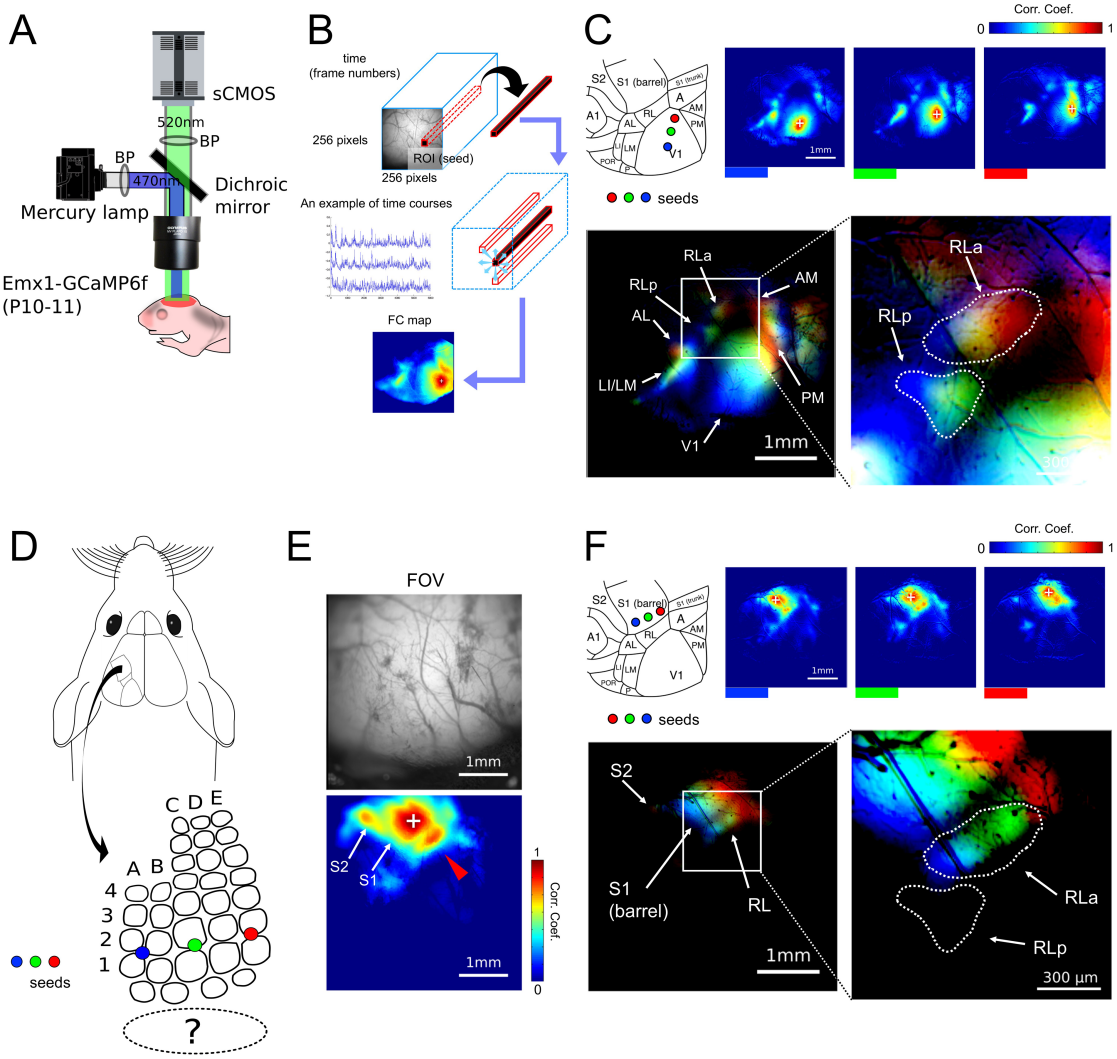
Retinotopy-like and somatotopy-like patterns of ongoing activity in developing mice. **(A)** Experimental setups for wide-field calcium imaging of developing mice (P10-11). Mice were awake during the recording of spontaneous cortical activity. **(B)** A schema of functional connectivity (FC) analysis. First, the ROI (seed) that consists of 1 × 1 pixel was arbitrarily picked up within the FOV. Then, the Pearson correlation coefficients were calculated between the dF/F time course of the extracted seed and the time courses of all other pixels to create FC maps. **(C)** FC maps obtained by putting the seeds in V1 along the upper-lower axis (blue, green, and red) and the merged retinotopy-like patterns. The right inset is the magnification of the left merged map around RLa and RLp within the white rectangle. The dashed lines in the right inset were drawn manually and indicate the areal borders of RLa and RLp. Scale bar: 1 mm, Scale bar in the inset: 300 μm. **(D)** A schema of seed positions in S1 barrel cortex. Seeds were put in S1 from blue, green, to red from lateral to medial, corresponding to the upper-lower axis. **(E)** An FC map obtained by putting a seed in the S1 barrel region and its FOV. Scale bar: 1 mm. **(F)** FC maps obtained by putting the seeds in the S1 barrel region along the upper-lower axis (blue, green, and red) and the merged somatotopy-like patterns. The right inset is the magnification of the left merged map around RLa and RLp within the white rectangle. The dashed lines in the right inset are identical to those in Figure 4C. Lines were drawn manually and indicate the areal borders of RLa and RLp based on the retinotopy-like patterns in **C**. Scale bar: 1 mm, Scale bar in the inset: 300 μm.

So far, we found that RL was divided into two subregions, RLa and RLp in adult mice (Figure 1). Importantly, only RLa has a topographic match between retinotopy and somatotopy along the upper-lower axis in the stimulus space (Figure 2). Next, we investigated the development of the segregation of RLa and RLp, and the topographic match between retinotopy and somatotopy. To test whether these structures are formed without spatially matched multimodal sensory inputs, we observed ongoing cortical activity in the developing mouse cortex before eye opening [postnatal day (P) 10–11] using wide-field imaging (Figure 3A). Since it is known that S1 barrel region responds to whisker stimulation in neonatal mice (Cai et al., 2022), it is hard to dissociate intrinsic spontaneous cortical activity and response to whisker stimulation. Although we do not expect whiskers to contact some objects during the image acquisition, in this section we refer to the cortical activity in somatosensory cortices as “ongoing activities.”

First, to test whether the segregation of RLa and RLp is identifiable in developing mice before eye-opening, we performed functional connectivity (FC) analysis (Matsui et al., 2016; Figure 3B). In FC analysis, we put the seed (arbitrary picked-up ROI) within V1 and made FC maps by calculating the correlation coefficients of calcium signal change between the seed and all the other pixels (Figures 3B,C upper panels). When we put seeds on three points in V1, which roughly corresponded to the upper to the lower visual field (Figure 3C), the shift of highly correlated spots was observed in RLa and RLp at P10-11 mice (Figure 3C). Based on these retinotopy-like patterns (Murakami et al., 2022), we found that RLa and RLp were already segmented at P10-11 (Figure 3C bottom). Thus, we could identify RLa and RLp based on the retinotopy-like structure of spontaneous activity before eye-opening.

It has already been reported that retinotopy-like patterns can be identified by FC analysis of spontaneous activity before eye opening (Murakami et al., 2022). However, it remains to be known whether somatotopy-like patterns in non-S1 areas can be observed in developing mice by FC analysis of ongoing cortical activity. Therefore, we next performed FC analysis again by putting the seed within the S1 barrel region (Figure 3D). We found high correlation spots in areas surrounding the S1, which corresponded to regions of a secondary somatosensory area (S2) and RL (Figure 3E). Furthermore, when we put the seeds in three different positions in S1, which corresponded to roughly the upper to the lower whiskers (Figure 3D), the highly correlated spots around S1 shifted according to the seed positions (Figure 3F). We used six mice, and in five of them we observed retinotopy-like structures in RLa and RLp and somatotopy-like structures in RLa described above (Supplementary Figure 5). One mouse was excluded from the entire analysis because we could not clearly segregate between RLa and RLp in the FC analysis, possibly due to the low spatial resolution of wide-field imaging. These results indicate that ongoing activity-based somatotopy-like patterns are already established at P10-11.

### 2.4 Overlap of topographic alignment between retinotopy-like and somatotopy-like patterns in RLa before eye-opening

To observe the overlap of retinotopy-like and somatotopy-like patterns of ongoing activity, we manually defined the regions of RLa and RLp based on retinotopic-like structures of the FC map (Figure 3C), and overlaid them on the FC map of the somatotopy-like patterns, in which seeds were put in the S1 barrel along the upper-lower axis of the whisker array (Figure 3F). We found that RLa, but not RLp, mainly overlapped somatotopy-like patterns: the color order from blue to green to red roughly corresponds to the upper to the lower receptive fields in both visual and whisker somatosensory systems (Figures 3C,F). These results show that a whisker-related somatotopy-like pattern is embedded in the cortical ongoing activity of P10-11 developing mice, and suggest that RLa has a somatotopic-like structure before eye opening.

**Figure 4:**
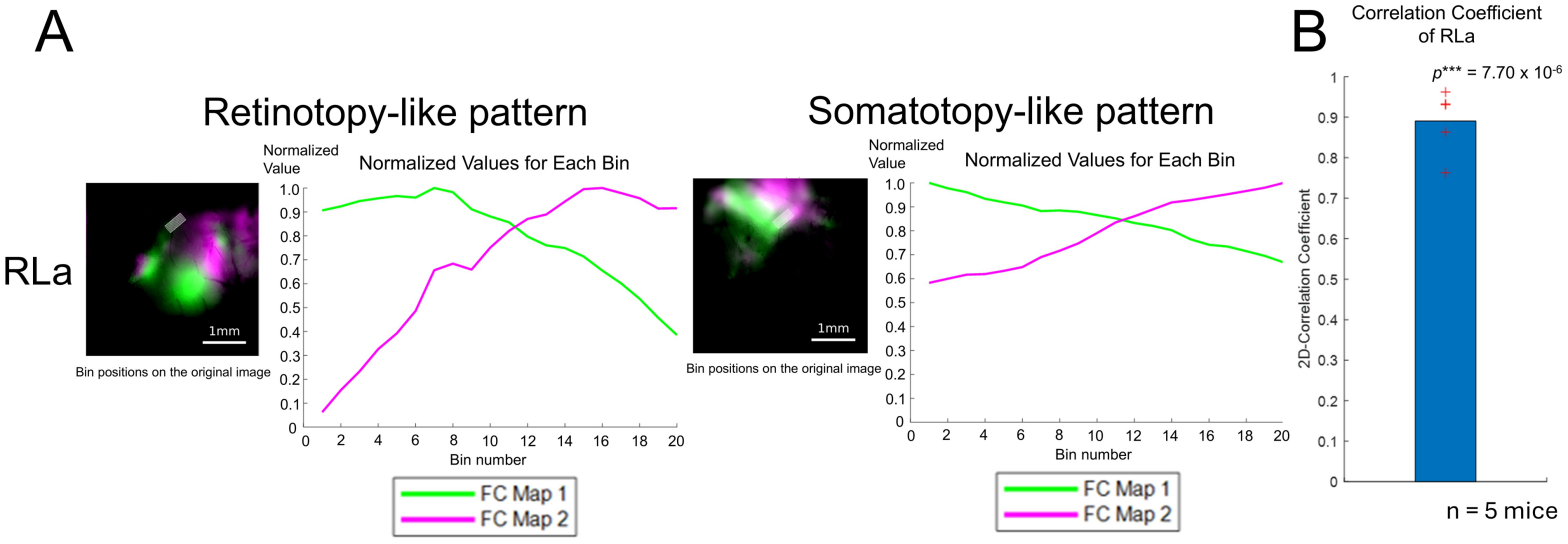
Quantification of topographic correspondence between retinotopy-like and somatotopy-like patterns in RL before eye-opening. **(A)** Quantification of topographic correspondence between retinotopy-like and somatotopy-like patterns in RLa. As performed in adult mice, the ROIs for RLa and RLp were segmented into 20 bins along the upper-lower axis (i.e., the longer side of the parallelograms). Bin positions are shown on top of the merged response maps. Scale bar: 1 mm. Within each bin, the normalized dF/F was calculated for each map corresponding to green and magenta channels respectively, by dividing the original dF/F with the absolute peak value. The x-axis indicates the bin number. Bin number from 1 to 20 corresponds to the upper to lower in the axis. The y-axis indicates the normalized dF/F. Green and magenta correspond to the response map with upper to lower visual stimuli. **(B)** 2D-correlation values of RLa for *n* = 5 mice. The red cross marks represent the 2D-CC from each mouse. RLa showed a 2D-CC that was significantly higher than zero (0.891 ± 0.0799) (mean ± standard deviation (SD); one-sample one-sided *t*-test; *p* = 7.70 × 10^−6^ < 0.025). ***p-value smaller than the significance level at 0.001.

To quantify the topographic alignment in developing mice (Figures 3C,F), we obtained profiles of retinotopy-like and somatotopy-like structures of ongoing activity using the same procedure (see Methods) as in Figure 1E, and calculated their correlation coefficient as we performed for the response profiles in adults (Figures 4A,B). We calculated 2D-CC between normalized dF/F values of the retinotopy-like and somatotopy-like structures in RLa (Figure 4A). 2D-CC of RLa was revealed to be significantly higher than zero (0.891 ± 0.0799) (mean ± standard deviation (SD); one-sample one-sided *t*-test; *p* = 7.70 × 10^−6^ < 0.025) (Figure 4B). These results suggest that rough topographic alignment of retinotopy-like and somatotopy-like patterns exists in RLa along the upper-lower axis in the stimulus space before the onset of visual input.

## 3 Discussion

We investigated whether topographic organizations between retinotopy and somatotopy existed in the rostrolateral area (RL), one of the higher visual areas (HVAs) in mice. By performing retinotopic and somatotopic mapping with wide-field calcium imaging, we found that area RL is divided into two distinct subregions, RLa and RLp, based on the retinotopic map (Figure 1), and revealed that only RLa, but not RLp, had somatotopy, which had topographic correspondence with retinotopy (Figure 2). We further revealed that RLa and RLp responded to whisker stimulation in a different way (Supplementary Figure 2). Furthermore, we examined how the topographic organization of ongoing activity is formed during development with functional connectivity (FC) analysis and found that the rough alignment between retinotopy-like and somatotopy-like structures in RLa already existed at postnatal (P) 10–11, before eye-opening (Figures 3, 4).

### 3.1 Segregation of RLa and RLp in mouse HVAs

The areal parcellation of HVAs is still controversial, and as far as we know, this is the first study reporting the existence of subregions in RL, namely, RLa and RLp. Many studies have been conducted to identify the areal boundaries of HVAs in mice, both anatomically (Wang and Burkhalter, 2007) and functionally (Garrett et al., 2014; Zhuang et al., 2017). Previous functional studies obtained a retinotopy map using moving checkerboard bar stimuli across the entire visual field and determined the borders of HVAs. However, especially in the anterior HVAs, the borders of the areas became blurred, making it difficult to clearly identify HVAs as defined by anatomical tracing studies. On the other hand, anatomical studies did not clearly determine areal borders; instead, they identified the position of HVAs based on the retinotopic structure of axonal projections from V1 to HVAs by injecting anterograde tracers into three locations within V1. This allowed for clear differentiation of the three anterior HVAs, RL, A, and AM.

Following this anatomical method, the present study used drifting grating stimuli through small apertures, which elicit spot-like response patterns, not to define areal boundaries, but to identify the retinotopic structure of each HVA. As a result, we were able to confirm through functional imaging that RL and A are distinct areas, and we further discovered two distinct regions within RL that have independent retinotopic organizations (Figure 1). Although this structure was not mentioned in the text of the previous anatomical study, an intricate retinotopic structure was observed within RL (Figure 4B in Wang and Burkhalter, 2007). Thus, while clear identification of the boundaries between higher visual areas remains challenging, we demonstrated the correspondence between the anatomical and functional determination of HVAs and further demonstrated the segregation of RLa and RLp within RL.

### 3.2 Topographic correspondence between different modalities

RL is located between V1 and S1 and is known to receive inputs from both V1 and S1 (Wang et al., 2012; Olcese et al., 2013; Hovde et al., 2019; Gilissen et al., 2021). A previous study showed that RL processed both visual and whisker somatosensory information (Olcese et al., 2013). Considering that RL preferentially represents a rostral part of the visual field close to the mouse (La Chioma et al., 2019), possibly overlapping with whisker representational space, it is plausible that RL is involved in processing visuo-tactile spatial congruence, an idea further supported by the present study.

In the present study, we showed that in RLa, a rough topographical correspondence exists between retinotopy and somatotopy along the upper-lower axis (Figure 2). While Olcese et al. (2013) report that RL has spatial segregations for visual and whisker response along the upper-lower axis, they take the upper-lower axis of the whisker pad along the temporal-nasal direction of mice, corresponding to the axis of the barrel columns (Figure 1F in Olcese et al., 2013). In contrast with this, the present study took the upper-lower axis along the dorsoventral direction of mice, corresponding to the axis of the barrel rows (Figure 2A), based on the previous literature (Figure 1 in Petersen, 2019). A recent study of a reconstructed 3D model of whisker tip positions (Supplementary Figure 1J in Weiler et al., 2024) demonstrated that shifting across the barrel columns (e.g., from B1 to B2 whiskers), as well as shifting across the barrel rows (e.g., from B2 to C2 whiskers), can be the moving along the upper-lower (elevation) axis in the visual field to some extent. Thus, it is possible that Olcese et al. (2013) saw the effect of upper-lower axis segregation, although the way they set the upper-lower axis is orthogonal to the present study, and shifting row by row as the present study did is closer to the vertical movement (Weiler et al., 2024). Also, the topographic correspondence observed in both the present study and Olcese et al. (2013) is not a precise matching of visual and tactile receptive fields, but a rough alignment of their upper-lower axis. Having an approximate, not-too-accurate upper-lower multimodal correspondence can be reasonable, considering that mice move their whiskers in a way such as protraction and retraction (Petersen, 2019) and that mice move their eyes as well (Stahl, 2004). Overall, topographic correspondence between retinotopy and somatotopy (Figure 2) and the response to whisker stimulation in RLa (Supplementary Figure 2) shown in this study suggest that cortical processing in RLa plays an important role in the spatial integration of visual and somatosensory information.

The topographic correspondence exists in other species, including primates and humans. In monkeys, visual and tactile receptive fields are spatially aligned in the ventral intraparietal area (VIP) (Duhamel et al., 1998). Topographical organizations of visuotactile maps in the area homologous to primate VIP were also shown in a human fMRI study (Huang et al., 2017). Thus, topographical organization between visual and tactile maps in associative cortices might be a common structure preserved in mammals across species.

### 3.3 Initial formation of the rough topographic correspondence across modalities during development

The sensory information processing pathways in the brain are formed by both intrinsic programs, such as molecular mechanisms and spontaneous neural activity during development, and extrinsic programs such as sensory inputs from the external environment. Previous studies have reported that topographic structures are formed by intrinsic programs, particularly spontaneous or ongoing activity from peripheral sensory receptors, in various sensory areas including the visual (Torborg and Feller, 2005; Cang et al., 2005b; Xu et al., 2011), somatosensory (Allène et al., 2008; Erzurumlu and Gaspar, 2012), and auditory cortices (Clause et al., 2014). However, it was unclear whether the alignment of topographic structures in sensory integration is formed by intrinsic or extrinsic programs. The present result revealed that the topographic alignment of visual and somatosensory modalities was already present in RLa before eye-opening as a pattern of ongoing cortical activity (Figures 3, 4), suggesting that the initial formation of topographic alignment is independent of spatially matched multisensory inputs. Although it is reported that external light can elicit glutamatergic retinal waves (Tiriac et al., 2018), it might be difficult for mice to associate external light through the eyelid, which contains less spatial information compared to after eye-opening, with particular whisker stimulation. Overall, while it is conceivable that spatially matched multisensory inputs refine the topographic correspondence at a later stage, the initial formation of topographic correspondence, as well as the topography within each modality, can be organized by intrinsic brain programs.

Intrinsic programs include spontaneous or ongoing neural activity and molecular control. Since corticocortical projections from V1 to HVAs are formed with the help of retinal spontaneous activity (Murakami et al., 2022), it is plausible that the pattern of spontaneous or ongoing activity with retinotopic and somatotopic structures in RLa may play a similar role in the formation of topographically corresponding corticocortical projections from V1 and S1 to RLa.

On the other hand, it is possible that projections from S1 to RLa are induced through molecular control, forming a matching topographic structure. In the visual pathway from the retina to V1, the Eph/ephrin family plays a crucial role in guiding axons to their appropriate targets, which is essential for the initial formation of retinal topography (Cang et al., 2005a). Recent studies have shown that postnatal *SOX11* expression levels regulate the separation of projections from S1 to S2 or M1 (Klingler et al., 2021), showing that the formation of corticocortical connections is controlled by molecular expression. Thus, both spontaneous or ongoing neural activity and molecular control remain possible mechanisms by which the topographic alignment of RLa is formed.

How is the pattern of spontaneous activity with roughly aligned retinotopic and somatotopic structures propagated to RLa? Spontaneous activity encoding retinotopic structure is derived from the retina (Torborg and Feller, 2005; Cang et al., 2005b; Xu et al., 2011), and cortical ongoing activity with whisker somatotopic structure may be generated by neurons in the whisker pad (Martini et al., 2021). Previous studies have shown that HVAs, including RLa, receive neural activity from lateral posterior nucleus (LPN) before corticocortical connections from V1 are established (Murakami et al., 2022). Furthermore, in adults, RL receives projections not only from LPN but also from the posterior thalamic complex (PO), which are higher-order somatosensory thalamic nuclei. Ongoing activity in RL a few days after birth persists even when retinal activity is eliminated (Murakami et al., 2022). These findings suggest that RL may receive neural activity from both visual and somatosensory pathways at this stage, although it has not yet been investigated whether ongoing somatosensory activity at this stage is propagated from PO to RLa.

Although the present study revealed that the initial rough topographic alignment does not require the spatially matched multisensory inputs, several problems still remain. Anatomically, it has not yet been investigated when corticocortical projections from S1 to RLa are established. Furthermore, the mechanisms by which retinotopic and somatotopic activity patterns, which propagate to the RLa through different pathways, are roughly aligned within the RLa are completely unknown. If the parallel projections from LPN and PO to RLa convey the retinotopic and somatotopic activity patterns as described above, and their topographies are roughly aligned, there should be some mechanisms to support the alignment of topographies of these parallel thalamocortical projections. If retinotopic and somatotopic activity patterns are generated in the periphery and propagated to the cortex, these activity patterns should be independent of each other, and there should be no topographic correspondence. Then, it is difficult to assume that neural activity causes the corresponding topographies of the parallel thalamocortical projections from LPN and PO to RLa, and they may rely on molecular control. Alternatively, if there are multimodal subcortical nuclei that send projections to RL, and if the visual and somatosensory topographies are already aligned there, neural activity consistent with their topographies could propagate from there to RL. Elucidating these mechanisms could provide insights to understand the development of neural circuits for unified multimodal perception.

## 4 Materials and methods

### 4.1 Animals

*Thy1-GCaMP6s* (GP4.3D; Jax stock no. 024275; Chen et al., 2013; Dana et al., 2014), *Emx1*-IRES-*cre* (Jax stock no. 005628; Gorski et al., 2002), and *Rosa*-CAG-LSL-*GCaMP6f* (Ai95; JAX stock no. 024105; Madisen et al., 2015) transgenic C57BL/6 J mice were obtained from the Jackson Laboratory. Mice were housed on a 12-h light/12-h dark cycle (temperature, 21–26°C; humidity, 40–70%).

Adult male and female Thy1-GCaMP6s mice (2–4 months) were used for *in vivo* wide-field and two-photon calcium imaging. We used 12 adult mice for *in vivo* functional imaging. To record the spontaneous activity of developing mice, we crossed *Emx1*-IRES-*cre* and Ai95 mice to obtain the transgenic mice expressing GCaMP6f in the cortical excitatory neurons. 6 male and female mice (P10–11, before eye-opening) were used, and one mouse was excluded from the entire analysis because we could not clearly segregate RLa and RLp in functional connectivity (FC) analysis.

All experiments were performed according to the institutional animal welfare guidelines laid out by the Animal Care and Ethical Committee of the University of Tokyo and approved by the Animal Experimental Committee of the University of Tokyo.

### 4.2 Surgical procedures

During the surgery, adult mice were anesthetized with 3% isoflurane for induction and 1–2% isoflurane for maintenance. After the scalp was excised and the surface of the skull was revealed, a custom-made metal head plate was attached to the skull using dental cement. Subsequently, a 4 mm-diameter cranial window was created over the left visual cortex (central position: 1 mm anterior and 3 mm lateral from the lambda). Durotomy was performed, and the aperture was sealed with a glass coverslip.

As for the developing mice, anesthesia was induced and maintained during the surgical procedure under 3% and 1–2% isoflurane, respectively. Prior to surgery, Xylocaine jelly (AstraZeneca) was applied to the skin. A custom-designed metal head plate was applied to the skull with dental cement. To maintain transparency, the skull over the cortex was kept moist and sealed with 1% agarose dissolved in artificial cerebrospinal fluid (ACSF; containing 150 mM NaCl, 2.5 mM KCl, and 10 mM HEPES at pH 7.4) along with a glass coverslip. After surgery, the mice were left in a cage for 6–10 h to recover from anesthesia. During the imaging session, body temperature was maintained at 37°C using a heating pad.

### 4.3 Stimulation protocols

#### 4.3.1 Visual stimulation

The visual stimuli were presented on a 32-inch LCD monitor using PsychoPy 2 (Peirce et al., 2019) or 3. The monitor was set in front of the right eye of the mouse in a way that the distance between the eye and the center of the screen was around 18 cm when measured orthogonally from the screen.

For retinotopic mapping, drifting circle grating stimuli with a size of 20 degrees in diameter, spatial frequency (SF) of 0.02 cycles per degree (cpd), and temporal frequency (TF) of 4.0 Hz. The visual stimuli were presented sequentially at nine locations on the monitor (arranged in a 3 × 3 grid). At each position, the drifting grating stimulus was presented for 2 s after 4 s of a gray screen. The stimuli were repeated 10 times.

For the multimodal experiments in wide-field calcium imaging, grating stimuli that drifted from left to right were presented at the center of the monitor with a size of 40 degrees in diameter, SF and TF being 0.02 cpd and 4.0 Hz, respectively. Each trial consisted of a 4-s baseline (gray screen), a 2-s stimulus of each direction, and a 2-s blank (gray screen). The stimuli were presented 20 times per direction.

#### 4.3.2 Whisker stimulation

Whiskers on the right side of a mouse were trimmed so that the whiskers that correspond to A1–A3, B1–B3, C1–C3, D1–D3, and E1–E3 in the barrel cortex (Petersen, 2019) were left. Two to three whiskers on the right side were stimulated by a 10 cm wire which was attached to a piezoelectric bimorph bender (Thorlabs, PB4NB2S). Piezo bender vibrated at 50 Hz. Two seconds stimuli were applied with a 6-s blank. PsychoPy3 (Peirce et al., 2019) was used to manage the stimuli. The protocol of whisker stimulation was the same for two-photon calcium imaging.

For multisensory stimuli, the visual stimuli and whisker stimuli described above were applied at the same time (6-s baseline and 2-s stimuli) during wide-field or two-photon calcium imaging.

### 4.4 *In vivo* calcium imaging

#### 4.4.1 Wide-field calcium imaging

Wide-field calcium imaging was performed for adult mice during the presentation of visual, whisker, and multisensory stimuli. The imaging was performed using macro-zoom fluorescence microscopy (MVX-10, Olympus) which was equipped with 1 × objective (MV PLAPO, Olympus), a mercury lamp (U-HGLGPS, Olympus), a GFP filter set (GFP mirror unit, U-MGFPHQ/XL; Olympus; excitation wavelength: 470 nm, emission wavelength: 520 nm), and an sCMOS camera (Andor Zyla 4.2, Oxford Instruments). Images were captured at a frame rate of 5 Hz with readily available software (NIS Elements, Nikon). During the recording, mice were sedated with 1.0–2.0 mg/kg chlorprothixene and weakly anesthetized with 0.2% isoflurane.

For imaging the spontaneous activity of developing mice, we used the same microscopy settings as adult mice. GCaMP6f was excited with a mercury lamp (470 nm wavelength). Ca^2+^ signals were collected with 520 nm wavelength. The image was obtained at a 5 or 10 Hz frame rate for 20–30 min. Mice were awake state without anesthesia during the imaging.

#### 4.4.2 Two-photon calcium imaging

We acquired the excitatory neuronal activity from cortical layer 2/3 (depth: around 220, 245, and 272 μm from pia) while presenting whisker stimuli in the same way as wide-field calcium imaging. Two-photon microscopy (A1R-MP, Nikon) equipped with a 16 × objective (N. A. 0.80, CFI75 LWD 16X W, Nikon) was used for the recording. The excitation wavelength was 920 nm (Mai Tai, Deep See, Spectra-Physics). The image was acquired at around 4 Hz using a resonant scanner.

### 4.5 Data and statistical analysis

#### 4.5.1 Data analysis for adult mice

All analyses were conducted with customized codes using MATLAB R2018a and R2023b (MathWorks).

The calcium signal just 1 s before the start of stimulation was used as a baseline. The signal during the stimulus period was averaged and divided by the average of the baseline signal for both wide-field and two-photon calcium imaging (Figures 1B,C, 2D; Supplementary Figures 2A–D, 4B,C top; Figures 1B,C spatially Gaussian-filtered with sigma being 10). For two-photon calcium imaging data, vessel parts were masked. To show the time lapse in response to the whisker stimulation (Figure 2B), the signal at each frame was divided by the average baseline signal. After that, the figures were spatially smoothed by a Gaussian filter (sigma: 2.5) and normalized to display in the 0–9% signal change range. To visualize the retinotopic and somatotopic maps, brightness and contrast were manually adjusted on ImageJ 2.0.0-rc-49/1.51a software (NIH) (Schneider et al., 2012).

In the analysis of the functional difference between RLa and RLp (Supplementary Figure 2), the areal ROI was selected by combining the automatically drawn border using Otsu’s method (Otsu, 1979) and the manually drawn area with AND operation. When calculating dF/F for bar graphs (Supplementary Figures 2E–H) the signal intensity from 0.2 to 2.2 s after stimulus onset (image acquisition rate: 5 Hz) was used as a response period.

#### 4.5.2 Data analysis for two-photon calcium imaging data

Data analysis was conducted using MATLAB R2023b (MathWorks).

To obtain Supplementary Figure 4C (bottom) and Supplementary Figure 4D, first, a local correlation image was generated for each two-photon scan by calculating the Pearson correlation coefficients between each pixel’s time course and those of its immediate neighbors (3 × 3 window), then averaging these correlations to produce a single correlation value per pixel. Correlation images from A, B, and C row whisker stimulation trials in the same imaging plane were combined by taking the maximum correlation value at each pixel position. Next, template matching was performed on the correlation image using a difference of Gaussians filter to detect cells. Detected cells were manually adjusted. Time courses were extracted for each detected cell by averaging pixel intensities within cell boundaries. Neuropil contamination was corrected by subtracting 30% of the neuropil signal from each cell’s time course, where neuropil signals were extracted from ring-shaped regions (8-pixel radius) around each cell, excluding a 2-pixel buffer zone from all cell boundaries. In each whisker stimulation condition, cells were classified as responsive if the mean dF/F across trials was significantly different from zero in the *t*-test (*p* < 0.01, see also 4.5.5. Statistical analysis) and the amplitude of the mean dF/F was larger than or equal to 0.03 (3% dF/F increase). Lastly, response maps were generated by creating binary images, with pixels belonging to responding cells assigned a value of one. In Supplementary Figure 4D, all three layers for each whisker stimulation condition were overlaid.

#### 4.5.3 Analysis of spontaneous cortical activity of developing mice recorded by wide-field calcium imaging

Data analysis was conducted using MATLAB R2023b (MathWorks). The acquired data was preprocessed through a high-pass temporal filter (> 0.01 Hz) for functional connectivity (FC) analysis. We first selected the seed arbitrarily in each visual area to obtain FC maps. A seed was 1 × 1 pixel square. The seeds were placed in a way that the corresponding receptive field moves from the upper, the middle, and to the lower. Next, the time course was calculated for the selected seed. Lastly, the Pearson correlation coefficients (CC) of time courses between the seed and all the other pixels were computed and the computation result was shown as an FC map. The brightness and contrast of the retinotopy-like and somatotopy-like maps were adjusted by ImageJ 2.0.0-rc-49/1.51a software (NIH) (Schneider et al., 2012).

#### 4.5.4 Analysis for evaluating topographical organization of retinotopy and somatotopy

Data analysis was conducted using MATLAB R2023b (MathWorks). First, we selected parallelogram regions of interest (ROIs) for RLa and RLp, respectively, based on the retinotopic map. Only in adult mice, the retinotopy-based parallelogram ROIs were further converted to match the positions for the somatotopy field of view (FOV) based on vessel patterns. After obtaining and adjusting ROIs, each ROI was segmented into 20 bins along the upper-lower axis in the retinotopic map, i.e., the longer side of the parallelogram ROIs were segmented. Then, the average dF/F within each bin for both retinotopic and somatotopic maps was calculated. In adult mice, since retinotopic and somatotopic maps were obtained by merging three different response maps for stimuli in different receptive fields (upper to lower, respectively), we got two matrices for retinotopy and somatotopy in one area (RLa or RLp) which consist of 20 bin numbers × 3 channels (red, green, and blue) corresponding to each response map, respectively. As for the developing mice, we picked up two seeds for the analysis due to the limited retinotopy-like representation areas in FC in RL.

Next, we plotted the normalized values obtained by dividing dF/F by the absolute peak dF/F value in each channel. Lastly, the two-dimensional correlation coefficient (2D-CC) was calculated between normalized dF/F values of retinotopic and somatotopic maps to evaluate topographical organization. If there is a topographical overlap and correspondence between retinotopy and somatotopy, the CC value is expected to be near 1, meaning a positive correlation. On the other hand, if there was no correspondence between them, the CC value is expected to be around 0, meaning no correlation.

#### 4.5.5 Statistical analysis

MATLAB functions were used for all statistical analyses.

In the analysis for the evaluation of the topographic organization, a one-sample one-sided *t*-test was performed (*n* = 5 adult mice and *n* = 5 developing mice, respectively) to test if the 2D-CC between retinotopy (retinotopy-like structures for developing mice) and somatotopy (somatotopy-like structures for developing mice) of RLa was significantly higher than 0 or not. The value was considered significant if the *p*-value was less than 0.25. The adult and developing mice we used were different individuals.

Repeated measures ANOVA and *post hoc* Tukey’s HSD test were performed to compare the dF/F obtained by wide-field calcium imaging under 3 conditions (visual, whisker, and multimodal stimuli). The difference was considered significant if the *p*-value was less than 0.05.

To detect responsive cells in two-photon calcium imaging data, a *t*-test was used. The difference was considered significant if the *p*-value was less than 0.01.

## Data Availability

The datasets and codes used in this study are available from the corresponding authors upon reasonable request.

## Footnotes

^1^ https://connectivity.brain-map.org/3d-viewer
