# Supplementary material for: Topographic correspondence between retinotopic and whisker somatosensory map in mouse higher visual area and its development
Source: Front Neural Circuits. 2025 Sep 2;19:1552130. doi: 10.3389/fncir.2025.1552130 (PMC12436497; doi:10.3389/fncir.2025.1552130)
Supplement: Supplementary file 1 [file Data_Sheet_1.pdf]

Supplementary Material

Supplementary Figure 1

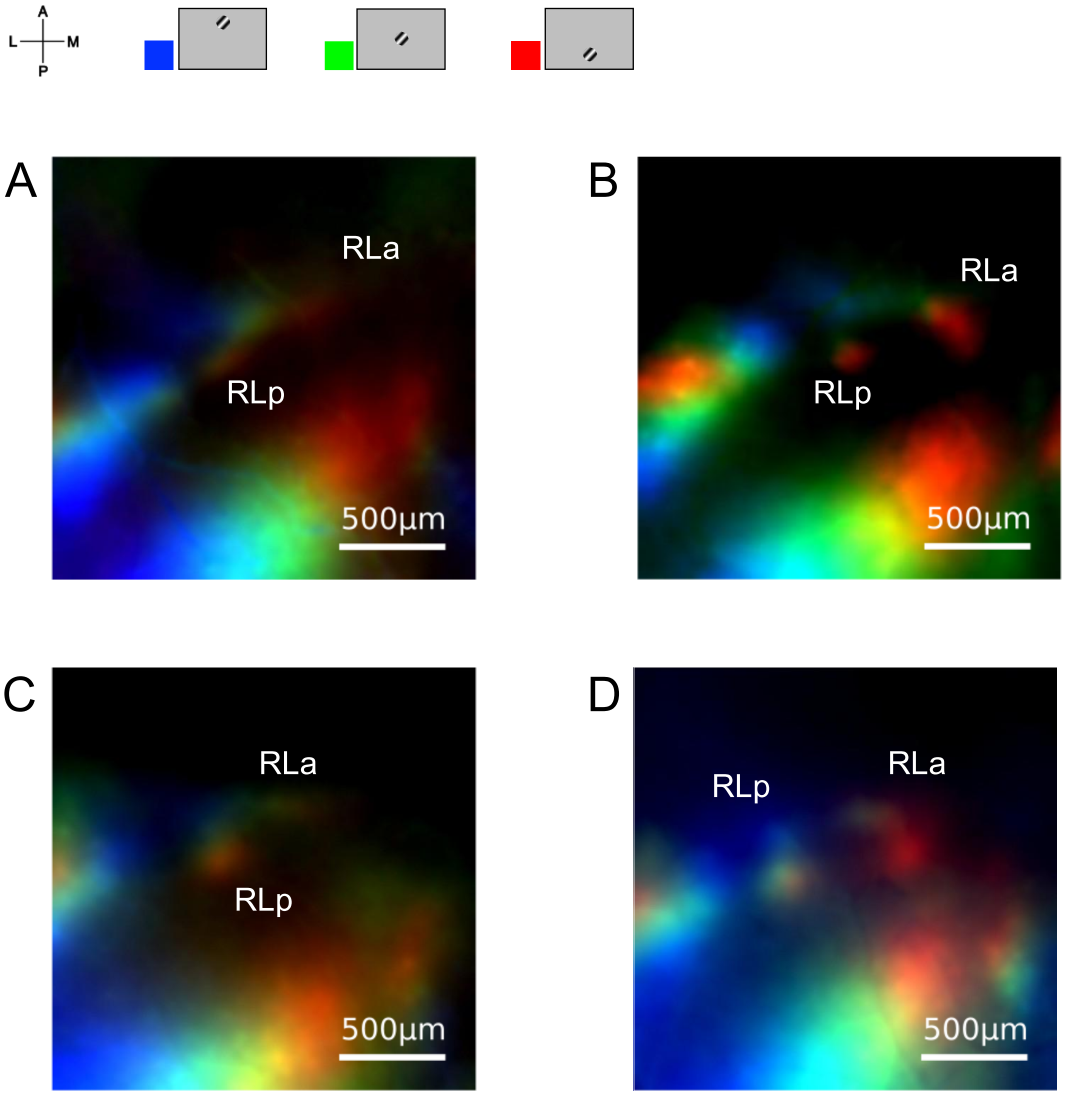

Supplementary Figure 1. Retinotopic maps of other mice reveal retinotopic structures in RLa and RLp.

(**A-D**) Retinotopic organization of visual areas for the other four mice used in the experiments. Color codes correspond to Fig. 1C and 1D. Scale bar: 500 µm.

Supplementary Figure 2

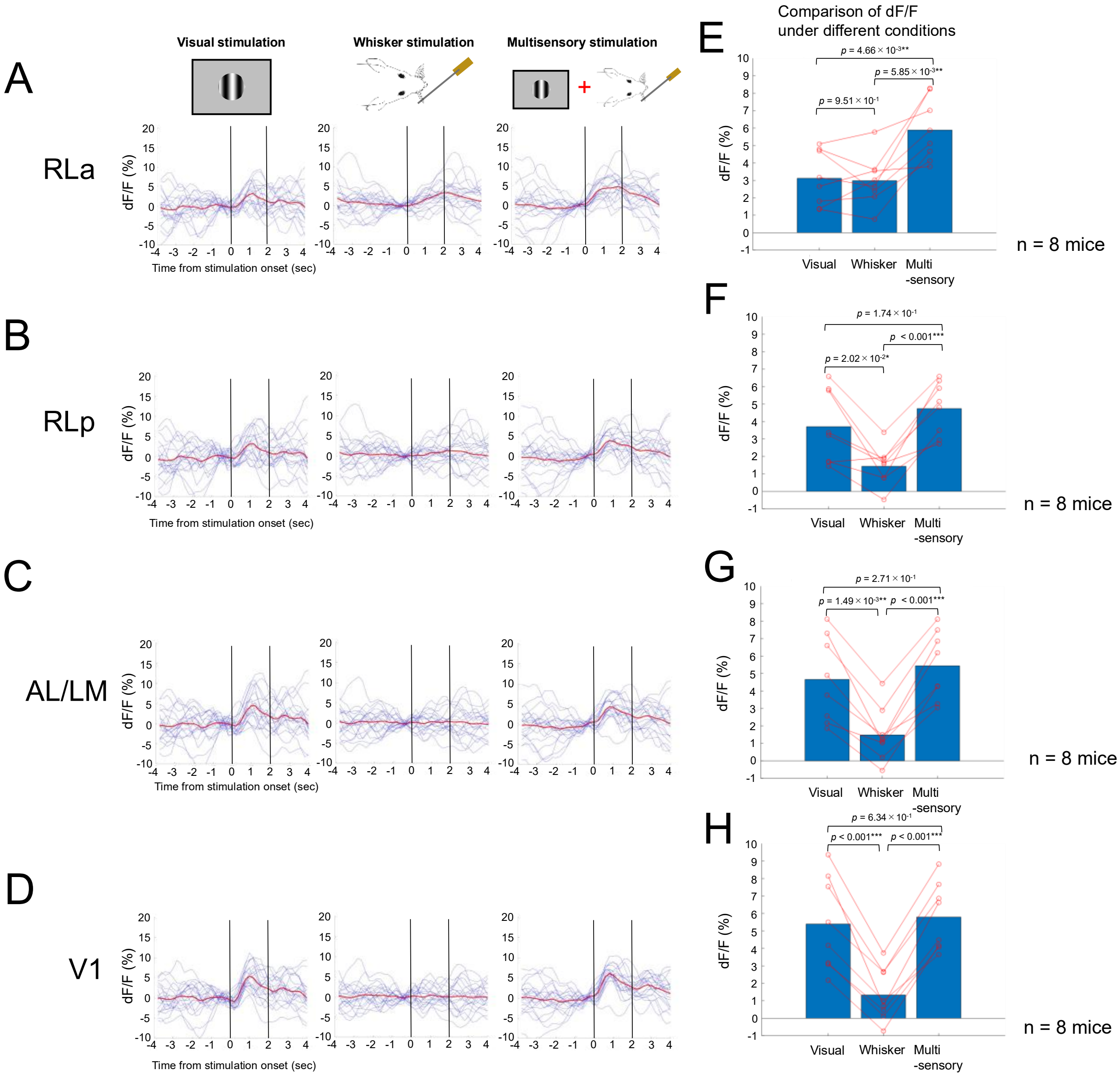

Supplementary Figure 2. Difference of functional properties of RLa and RLp in multimodal sensory processing.

(A-D) dF/F calcium signal change time courses under visual-only (left), whisker-only (middle), and multimodal (right) stimulation in RLa (A), RLp (B), AL/LM (C), and V1 (D). The black lines at 0 sec and 2 sec indicate 2-second stimulation onset and offset, respectively. The thick red line shows the average time course and the thin blue lines show the time courses of each trial (20 trials). The x-axes indicate the seconds (the onset of stimulation is set to 0) and the y-axes indicate the dF/F (%). Although all areas responded to visual stimuli, only RLa showed a clear response to both unimodal (visual or whisker) stimuli and a stronger response under the multimodal stimuli than unimodal stimuli. **(E-H)** Bar graphs that compare the response amplitude (averaged dF/F on the y-axis) between each stimulating condition in RLa (E), RLp (F), AL/LM (G), and V1 (H). The red circle

indicates the value of each mouse, and the data from the same mouse are connected with red lines. **(E)** RLa. Repeated-measures ANOVA and post hoc Tukey's HSD test were performed for the three conditions: unimodal visual, whisker, and multimodal stimuli (n = 8 mice). DF/F (%) = 3.12±1.57, 2.98±1.45, 5.89±1.78 (mean ± standard deviation (SD)) under visual, whisker, and multimodal conditions, respectively. Repeated-measures ANOVA: F (2,14) = 17.513, p < 0.0002; post hoc Tukey's HSD test. DF/F between visual and multisensory conditions are significantly different at the significance level of $p = 0.05$ with a p-value of 4.66×10^{-3}. DF/F between whisker and multisensory conditions are significantly different at the significance level of $p = 0.05$ with a p-value of 5.85×10^{-3}. DF/F between visual and whisker conditions are not significantly different at the significance level of $p = 0.05$ with a p-value of 9.51×10^{-1}. *: p-value smaller than the significance level at 0.05; **: p-value smaller than the significance level at 0.01; ***: p-value smaller than the significance level at 0.001. **(F)** RLp. Repeated-measures ANOVA and post hoc Tukey's HSD test were performed for the three conditions: unimodal visual, whisker, and multimodal stimuli (n = 8 mice). DF/F (%) =3.70±2.10, 1.43±1.12, 4.74±1.53 (mean ± SD) under visual, whisker, and multimodal conditions, respectively. Repeated-measures ANOVA: F (2,14) = 19.539, p < 0.0001; post hoc Tukey's HSD test. DF/F between visual and multisensory conditions are not significantly different at the significance level of $p = 0.05$ with a p-value of 1.74×10^{-1}. DF/F between whisker and multisensory conditions are significantly different at the significance level of $p = 0.05$ with a p-value 5.63×10^{-4}. DF/F between visual and whisker conditions are significantly different at the significance level of $p = 0.05$ with a p-value of 2.02×10^{-2}. *: p-value smaller than the significance level at 0.05; **: p-value smaller than the significance level at 0.01; ***: p-value smaller than the significance level at 0.001. **(G)** AL/LM. Repeated-measures ANOVA and post hoc Tukey's HSD test were performed for the three conditions: unimodal visual, whisker, and multimodal stimuli (n = 8 mice). DF/F (%) = 4.66±2.45, 1.48±1.55, 5.44±1.97 (mean ± SD) under visual, whisker, and multimodal conditions, respectively. Repeated-measures ANOVA: F (2,14) = 37.056, $p < 0.0001$; post hoc Tukey's HSD test. DF/F between visual and multisensory conditions are not significantly different at the significance level of $p = 0.05$ with a p-value of 2.71×10^{-1}. DF/F between whisker and multisensory conditions are significantly different at the significance level of $p = 0.05$ with a p-value of 1.46×10^{-4}. DF/F between visual and whisker conditions are not significantly different at the significance level of $p = 0.05$ with a p-value 1.49×10^{-3}. *: p-value smaller than the significance level at 0.05; **: p-value smaller than the significance level at 0.01; ***: p-value smaller than the significance level at 0.001. **(H)** V1. Repeated-measures ANOVA and post hoc Tukey's HSD test were performed for the three conditions: unimodal visual, whisker, and multimodal stimuli (n = 8 mice). DF/F (%) = 5.39±2.67, 1.33±1.52, 5.80±1.94 (mean ± SD) under visual, whisker, and multimodal conditions, respectively. Repeated-measures ANOVA: F (2,14) = 53.819, p < 0.0001; post hoc Tukey's HSD test. DF/F between visual and multisensory conditions are not significantly different at the significance level of $p = 0.05$ with a p-value of 6.34×10^{-1}. DF/F between whisker and multisensory conditions are significantly different at the significance level of $p = 0.05$ with a p-value of 1.05×10^{-5}. DF/F between visual and whisker conditions are significantly different at the significance level of $p = 0.05$ with a p-value of 7.53×10^{-4}. *: p-value smaller than the significance level at 0.05; **: p-value smaller than the significance level at 0.01; ***: p-value smaller than the significance level at 0.001.
There remains a possibility that, during multimodal stimulation, not only visual stimulation from the monitor but also the whisker stimulation apparatus itself works as another visual stimulation. However, the multisensory stimulation protocol was the same for all brain regions, and we observed significantly larger dF/F under the multimodal stimulation condition compared to both unimodal stimulation conditions only in RLa, while there is no significant difference between unimodal visual

and multimodal conditions in RLp, AL/LM, and V1. These results suggest that multisensory enhancement is one of the functional differences between RLa and RLp.

Supplementary Figure 3

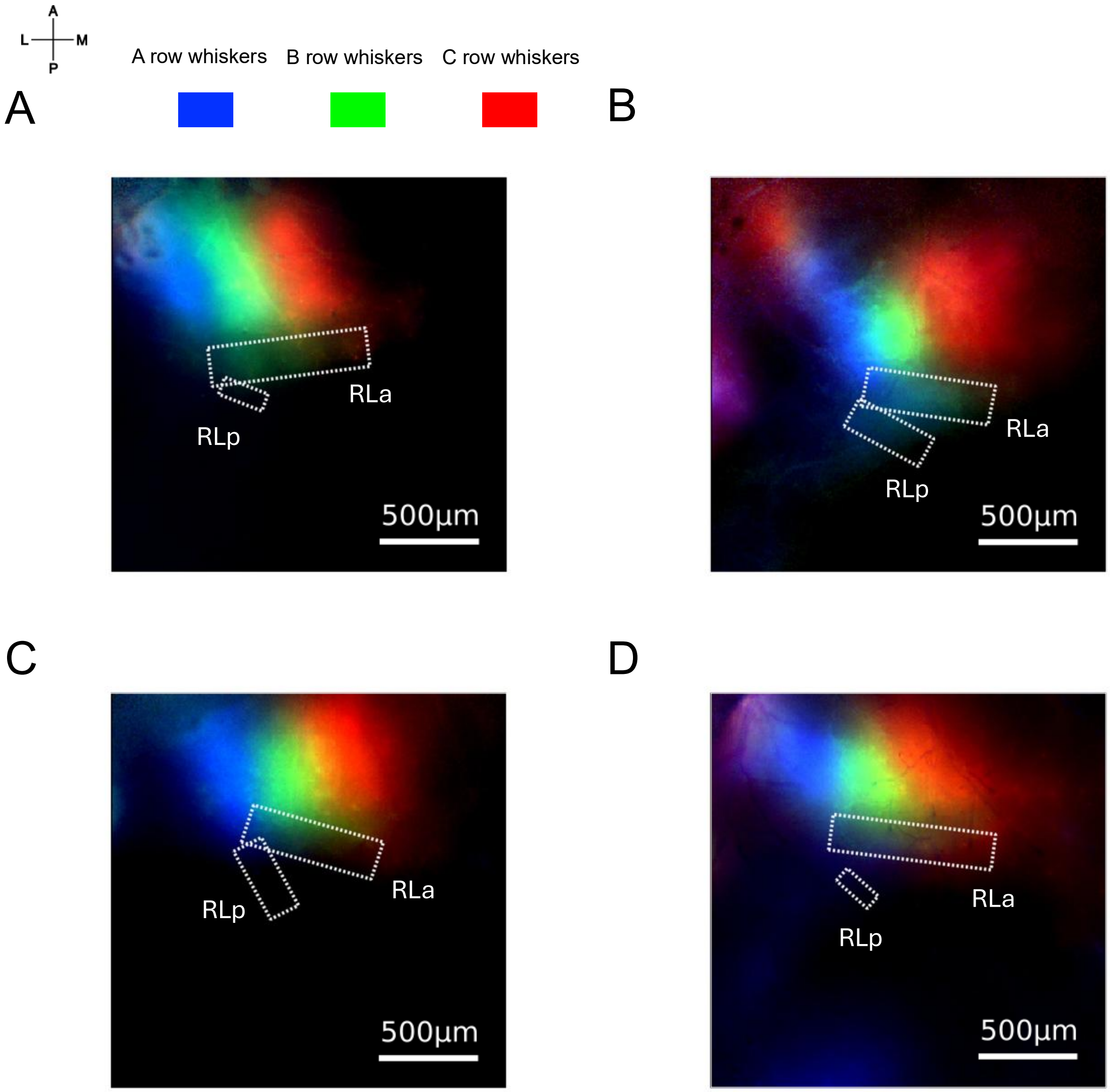

Supplementary Figure 3. RLa responds to A-C rows whisker stimulation in wide-field calcium imaging.

(A-D) Merged response maps to A, B, and C row whiskers stimulations in the other four mice used in experiments. During the response map acquisitions, the right eyelid of each mouse was sutured to avoid the effect of light as much as possible. Dashed white lines indicate the ROIs for RLa and RLp, which were identified based on the retinotopic maps. Scale bar: 500 µm.

Supplementary Figure 4

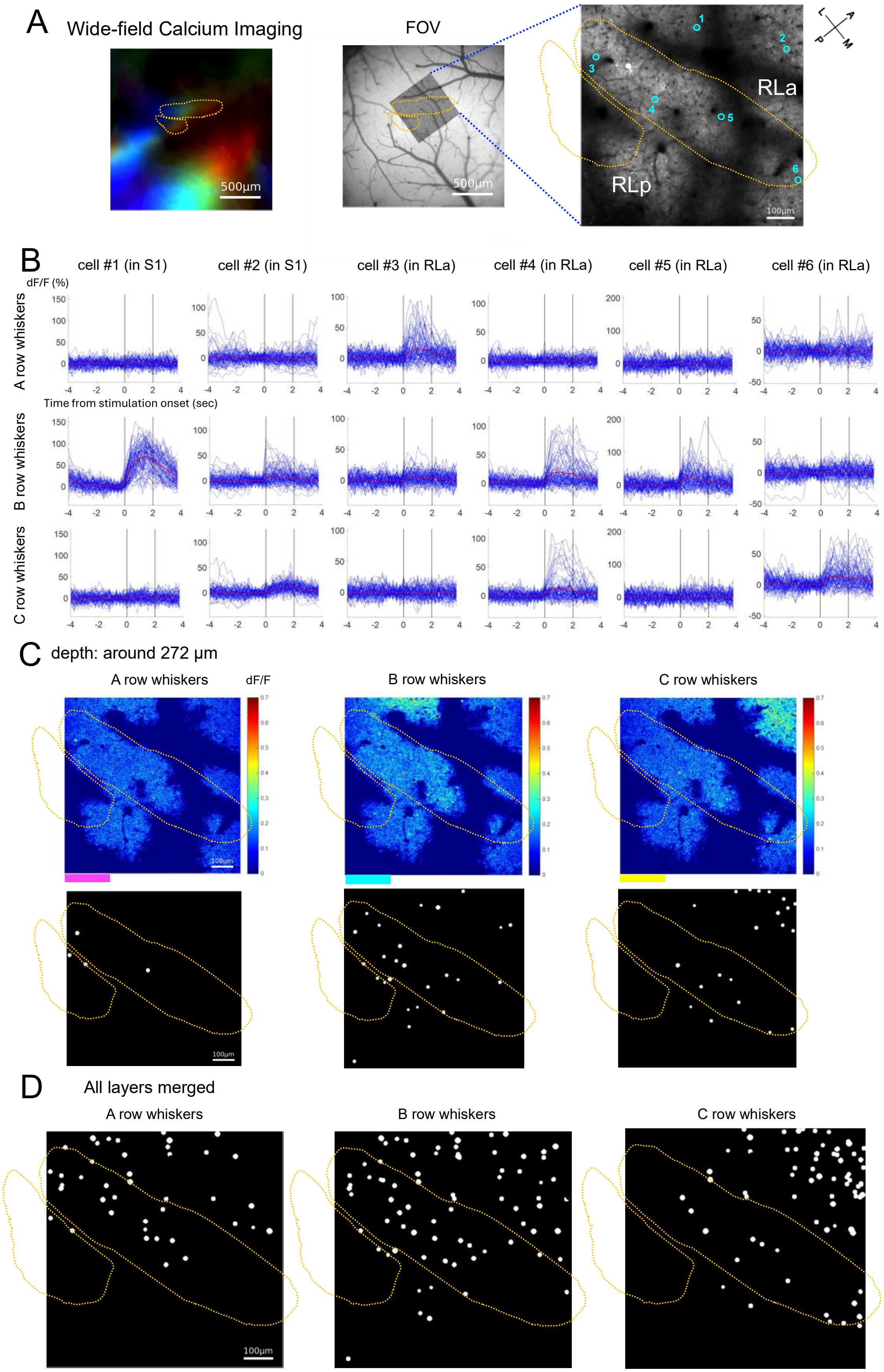

Supplementary Figure 4. Somatotopy in RLa obtained by two-photon calcium imaging.

(A) (Left) The original retinotopic map obtained in wide-field calcium imaging. The right eyelid was sutured to avoid the effect of light as much as possible. Dashed orange lines were manually created based on the retinotopic map indicating the ROIs of RLa and RLp. (Right) FOV of wide-field and two-photon calcium imaging with manually designated ROIs of RLa and RLp. The marked cells and their numbers correspond to the response time courses in Supplementary Figure 4B. Scale bar: 500 µm (Supplementary Figure 4A, FOV and the retinotopic map in macro-scale). Other scale bars: 100 µm. **(B)** Example time courses of A-C row whisker stimulation response at approximately 272 µm depth. The black lines at 0 sec and 2 sec indicate 2-second stimulation onset and offset, respectively. The thick red line shows the average time course and the thin blue lines show the time courses of each trial (80 trials). **(C)** (Top) Representative two-photon calcium imaging response maps to A, B, and C row whiskers (Bottom) Binarized response maps (see Methods) for each whisker stimulation condition at approximately 272 µm depth. Scale bars: 100 µm. **(D)** Binarized response maps for each whisker stimulation condition, overlaid across all three imaging planes (around 272 µm. 245 µm, and 220 µm depth). Scale bars: 100 µm.

Supplementary Figure 5

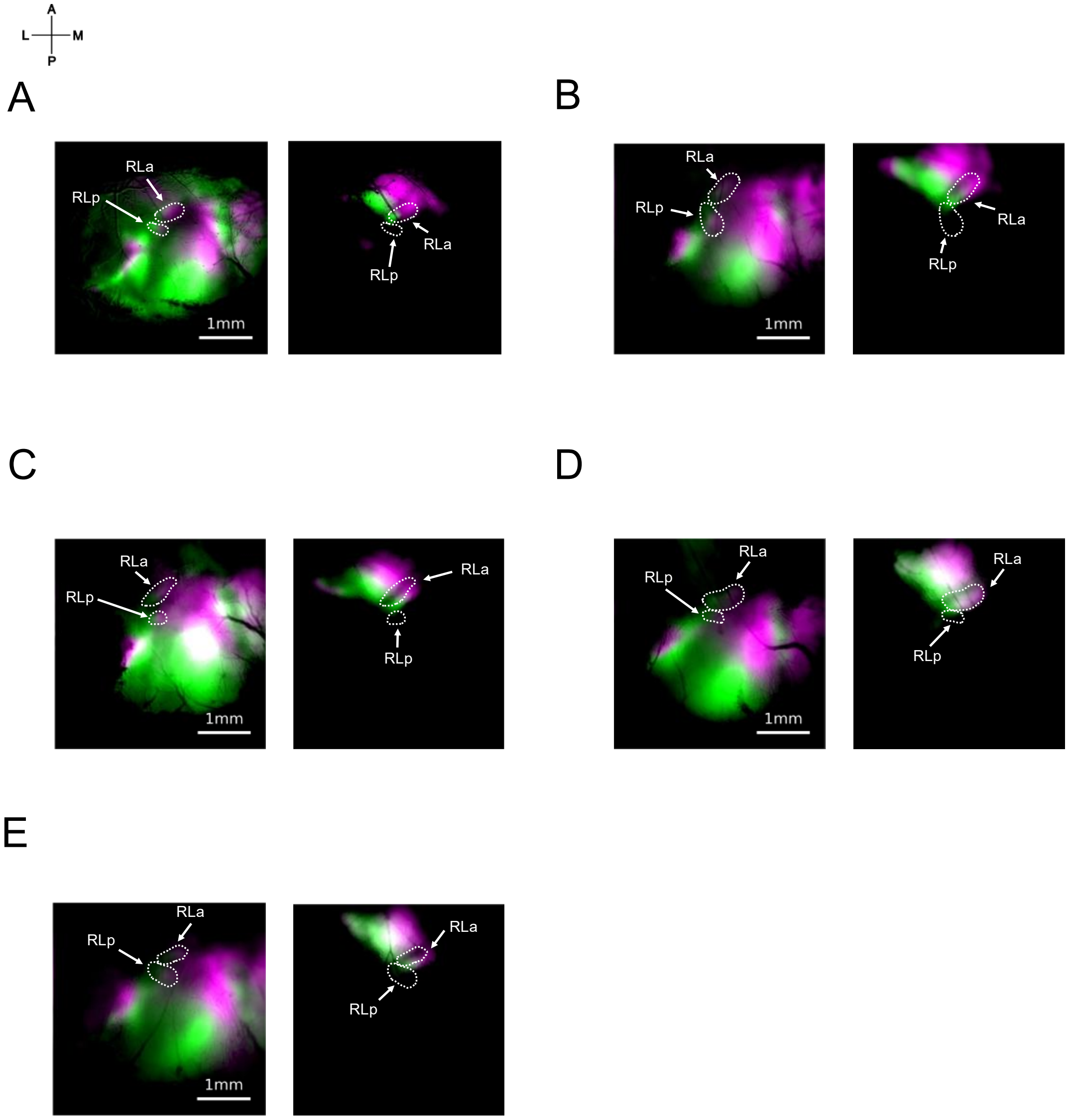

Supplementary Figure 5. Retinotopy-like and somatotopy-like patterns of other developing mice.

(A-E) Merged functional connectivity (FC) maps in five mice used in the analysis. (right) Retinotopy-like patterns obtained by putting two seeds in the primary visual area (V1). (left) Somatotopy-like patterns obtained by putting two seeds in the primary somatosensory area (S1). Dashed lines are the manually depicted boundaries of RLa and RLp based on the retinotopy-like patterns. Supplementary Figure 5A uses the same mouse as the data shown in Fig. 3C and 3F. Scale bar: 1mm.
